# The National School of Rehabilitation, Integration and Recovery in Mental Health- A case study of a unique academic and working model in the community

**DOI:** 10.1192/j.eurpsy.2023.699

**Published:** 2023-07-19

**Authors:** Y. Mazor, N. Hadas-Lidor, V. Baloush-Kleinman, O. Oren, S. Daass-Iraqi

**Affiliations:** ^1^Ono Academic college, Kiryat Ono; ^2^School of social work and welfare, Hebrew university of Jerusalem, Jerusalem, Israel

## Abstract

**Introduction:**

The National School of Rehabilitation, Integration and Recovery in Mental Health was established in 2011 at Ono Academic College. Its operation is supervised and funded by the Department of Rehabilitation in the Mental Health Division of the Israel Ministry of Health. The School offers courses for a broad variety of mental health professionals and others involved in the mental health field (service users and family members) to promote professional competencies, social cohesion, learning from experience, and to advance mental health rehabilitation. In recent years, the School has become increasingly involved in multiculturalism, providing special courses and workshops for both the Palestinian-Arab and ultra-Orthodox Jewish sectors in Israel. The School is based on the belief in the ability of service users to recover, integrate, and live meaningful lives in the community. This belief is consistent with values of the recovery approach (Slade et al., 2017). The School is constantly in dialogue with the community, and provides training for interventions that promote recovery, as well as social cohesion in the field of psychiatric rehabilitation.

**Objectives:**

To describe the process of foundation, implementation, and outcomes of the unique model of the School; to discuss the multicultural and social opportunities and challenges; to portray major elements of the school methodology and practice.

**Methods:**

Work model presentation through qualitative analyses of social and academical processes, alongside quantitative descriptive data.

**Results:**

Every year, 700 students from various helping professions in the field of psychiatric rehabilitation study at the School, as well as service users. The school operates as a bridge between academy and the field and encourages learner and staff diversity, joint learning, and discourse. Over 30 courses are conducted annually including evidence-based intervention courses such as IMR, and training courses such as knowledge by experience, supervision, rehabilitation coordinators, etc. In addition, unique courses are given, such as eating disorders, DBT, and social cognitive intervention training with multicultural adaptations. Lastly, the school offers consultation in implementing interventions within services.

**Conclusions:**

The working model of the School operates under the framework and values of recovery, social integration and cohesion, and multiculturalism. To this day, the School offers courses, symposiums, conferences, and professional publications, to educate for values of recovery and community inclusion, alongside improving the quality of services.

**Disclosure of Interest:**

None Declared

